# Recurrent Severe Hypothermia as a Manifestation of Central Thermoregulatory Dysfunction in a Patient With Cerebral Palsy and Shaken Baby Syndrome

**DOI:** 10.7759/cureus.102330

**Published:** 2026-01-26

**Authors:** Brooke Allnutt, Phillip Petrasko, Rafik Jacob

**Affiliations:** 1 Internal Medicine, University of Florida College of Medicine, Gainesville, USA; 2 Internal Medicine, University of Florida College of Medicine – Jacksonville, Jacksonville, USA; 3 Program for Adults with Intellectual and Developmental Disabilities, University of Florida College of Medicine – Jacksonville, Jacksonville, USA

**Keywords:** autonomic dysfunction, case report, central thermoregulation, cerebral palsy, hypothermia, shaken baby syndrome

## Abstract

Recurrent hypothermia is an uncommon but clinically significant problem in adults with prior traumatic brain injury (TBI), such as shaken baby syndrome (SBS). Central thermoregulatory dysfunction, due to hypothalamic or autonomic injury, can lead to episodic hypothermia that mimics endocrine pathology. We describe a 38-year-old woman with cerebral palsy (CP) and a remote history of SBS presenting with recurrent hypothermia despite normal thyroid and adrenal function. Her episodes were initially treated empirically with corticosteroids, given concern for adrenal involvement, but recurrent events prompted multidisciplinary evaluation. Multiple infectious, endocrine, and neurologic assessments were negative for secondary causes. Imaging confirmed microcephaly and encephalomalacia consistent with prior TBI. Hypothermia was managed with supportive warming and steroid tapering. Neurology and endocrinology attributed her symptoms to hypothalamic hypoplasia or autonomic dysfunction resulting from her early brain injury. This case highlights the importance of recognizing central hypothermia as a long-term sequela of SBS to prevent unnecessary corticosteroid exposure and to improve supportive management.

## Introduction

Non-accidental traumatic brain injury (TBI), such as shaken baby syndrome (SBS), can result in significant, permanent neurologic damage [1]. This is an infrequent diagnosis with an estimated prevalence of 25-30 cases per 100,000 infants under 1 year of age [2,3]. While motor and cognitive deficits are well-recognized, autonomic dysfunction is a less commonly discussed but critical sequela. The hypothalamus, which serves as the body's primary thermoregulatory center, is vulnerable to diffuse axonal injury and hypoxic-ischemic damage associated with TBI [4,5]. Damage to this region can impair the body's ability to perceive core temperature and mount appropriate physiological responses, leading to poikilothermia and a propensity for hypothermia [6]. We present a case of recurrent, severe hypothermia in an adult patient with severe cerebral palsy (CP) and a history of SBS, illustrating a clear etiology of central thermoregulatory failure.

## Case presentation

A 38-year-old woman with CP, SBS, microcephaly with lissencephaly, ventriculoperitoneal (VP) shunt, seizure disorder, blindness, gastroesophageal reflux disorder (GERD), glaucoma, osteoporosis, percutaneous endoscopic gastronomy (PEG)-tube dependence, and recurrent hypothermia presented for follow-up after a recent episode of hypothermia.

She is nonverbal, communicates with facial expressions, and resides in a group home with 24-hour caregiver support. Baseline functional status includes limited mobility and PEG dependence for nutrition. The patient's baseline temperature is chronically low, averaging 96.5°F. During acute episodes, her temperature drops significantly (e.g., 94°F during a home episode, 92°F during sepsis).

The patient's clinical course is marked by multiple hospital admissions for severe hypothermia.

In October of 2022, she was admitted for hypothermia of unknown origin. A sepsis workup revealed an unremarkable chest x-ray, urinalysis, and respiratory viral panel. A lumbar puncture was unsuccessful due to her anatomy from prior spinal fusion. She was treated empirically with vancomycin and cefepime for 10 days and eventually discharged home following resolution of her hypothermia.

In March of 2023, she was again admitted for septic shock secondary to a right lower lobe pneumonia. This admission was notable for profound hypothermia in the setting of infection with a temperature to 92°F, requiring active rewarming with a Bair Hugger (3M, China). Adrenal crisis was less likely the source of this hypothermia, as her serum a.m. cortisol was 37.2, and cosyntropin stimulation test was unremarkable. Respiratory viral panel and blood cultures were negative. She received intravenous fluid resuscitation, vasopressor support, and seven days of empiric antibiotics before being safely discharged.

In July of 2023, she was taken to a different hospital emergency department (ED) from prior admissions, this time seeking management of vomiting with suspected aspiration, abdominal distention, and constipation. She was initially treated for possible aspiration pneumonia, but antibiotics were discontinued due to low concern for sepsis. She was hypothermic and hypotensive without adequate response to intravenous fluid resuscitation and required ICU admission. Her blood pressure improved after receiving corticosteroid therapy, leading to concerns for adrenal insufficiency. Her hypothermia was attributed to chronic autonomic dysfunction, and she was discharged in stable condition.

In December of 2024, she was taken to the same ED from October 2022 and March 2023, this time seeking diagnosis and management following an episode of suspected aspiration pneumonia complicated by acute-on-chronic hypothermia. She was treated empirically with a course of antibiotics and sent home in stable condition on Augmentin.

In February of 2025, she returned to the same ED as previously evaluated, this time seeking diagnosis and management of hypothermia. Infectious evaluation was unremarkable. Radiographic evaluation with CT head was also unremarkable. Endocrinology was consulted and, following their diagnostic work-up, determined that adrenal insufficiency was an unlikely cause for her hypothermia. She was treated with an external warming device (Bair Hugger) and following return to her baseline temperature, was discharged home.

In March of 2025, she returned to the same ED as previously evaluated, this time seeking diagnosis and management of cough, hypertension, and altered mental status. She was hypotensive with adequate response to intravenous fluids and admitted for further evaluation of her abdominal distention. Chest x-ray was unremarkable. She started empiric antibiotics; however, following an unremarkable infectious work-up, antibiotic therapy was discontinued. Electroencephalograms (EEGs) did not reveal any epileptiform activity. She was diagnosed with adynamic ileus. Throughout her stay, she was hypothermic and treated with an external warming device (Bair Hugger). She returned to her baseline mental status and was discharged home on a corticosteroid taper. She had a follow-up appointment with endocrinology, who believed that her hypothermia was less likely related to an endocrine cause and recommended a neurology consultation and evaluation.

In June of 2025, she returned to the same ED as previously evaluated, this time seeking diagnosis and management of urinary retention. Her urinary retention resolved within 24 hours of presenting to the ED. While in the ED, her caregiver noted her temperature had much improved recently, so she was continued on the corticosteroid taper she was previously discharged on.

In October of 2025, she presented for routine follow-up to her internal medicine primary care provider. The caregiver reported a recent home episode of hypothermia managed with stress-dose hydrocortisone. Her endocrinologist advised her to taper and cease her corticosteroid therapy. Her caregivers desired to continue the corticosteroid therapy. Endocrine agreed to continue her on a morning corticosteroid dose with an additional pro re nata stress dose available for episodes of hypothermia. This visit also highlighted a concern for cyclical insomnia, where she would stay awake for two-three days, refractory to melatonin and conservative measures, followed by episodes of prolonged sleep.

After each hospitalization, the patient returned to her neurologic and thermal baseline after receiving a combination of intravenous resuscitation, vasopressors, empiric antibiotics, and active external rewarming with a Bair Hugger device. Her recurrent hypothermia continued to be treated with corticosteroid therapy, despite several endocrine assessments showing an intact hypothalamic-pituitary-adrenal (HPA) axis, likely contributing to her insomnia. Table 1 details the various etiologies that were possibly contributing to the patient's repeat hypothermia. The long-term management is focused on tapering the unnecessary corticosteroid therapy while managing her underlying central thermoregulatory dysfunction with supportive, non-pharmacologic measures (e.g., environmental temperature control, appropriate clothing, and temperature monitoring). The corticosteroid therapy was not indicated and was contributing to negative side-effects (i.e. insomnia), her primary care physician (PCP) prioritized functional sleep aids and coordinated corticosteroid taper and cessation.

**Table 1:** Etiology evaluation overview TSH: Thyroid stimulating hormone

Etiology	Considerations	Status
Sepsis/infection	Negative cultures; no systemic inflammatory response	Excluded
Adrenal insufficiency	Normal morning cortisol, stable vitals	Excluded
Hypothyroidism	Normal TSH	Excluded
Drug-induced (antiepileptics)	Not a common/known side-effect of any of the patient’s medications	Low likelihood
Central thermoregulatory dysfunction	Chronic pattern, hypothalamic hypoplasia	Most likely

## Discussion

This case illustrates the complex interplay between severe brain injury and autonomic dysfunction. The patient's history of SBS, resulting in microcephaly, encephalomalacia, and a VP shunt, provides an anatomical basis for central thermoregulatory failure. Another important consideration is that patients with severe CP often have compounding risk factors, including decreased muscle mass, impaired shivering response, limited voluntary control of clothing/environment, PEG dependence and malnutrition, reducing metabolic heat production, and chronic antiepileptic therapy modulating CNS neurotransmitters involved in temperature regulation [7-10].

The result of thorough neurology and endocrine evaluations is that her hypothermia likely stems from impaired thermoregulation, rather than an endocrine or medication-induced cause. Her brain's inability to properly regulate core temperature makes her more susceptible to environmental temperature changes. Her inability to regulate her core temperature allows for masking of typical signs of systemic illness, further complicating her care.

The management of this patient's hypothermia also presents a significant clinical challenge. The patient is nonverbal and unable to share her perspective directly. She relies on others to advocate for her well-being, as her mental baseline consists of limited nonverbal communication with facial expressions. The caregivers' observation of improvement with corticosteroid therapy, while subjectively valid, led to an empirical treatment regimen that was not supported by objective testing. This treatment, in turn, exacerbated her insomnia. This cyclical sleep deprivation further compromised her health, with caregivers noting an association between her insomnia cycles and an increased frequency of cold and flu-like symptoms. Her ongoing management correctly identifies the need to treat the insomnia by tapering her corticosteroid dose and focusing on supportive care.

## Conclusions

In patients with severe neurologic impairment from conditions like SBS, recurrent hypothermia should be investigated. When endocrine and infectious etiologies are excluded, impaired central thermoregulation secondary to hypothalamic damage should be considered as the primary cause. Management should focus on supportive care and environmental control. Empiric treatments should be used sparingly until further diagnostic work-up is completed as any treatment brings risks of iatrogenic side effects, as demonstrated by this patient’s insomnia exacerbated by corticosteroid use.
